# Supplementary material for: A WRKY Transcription Factor, EjWRKY17, from Eriobotrya japonica Enhances Drought Tolerance in Transgenic Arabidopsis
Source: Int J Mol Sci. 2021 May 25;22(11):5593. doi: 10.3390/ijms22115593 (PMC8197471; doi:10.3390/ijms22115593)
Supplement: Supplementary file 1 [file ijms-22-05593-s001.zip › ijms-1204590-supplementary.pdf]

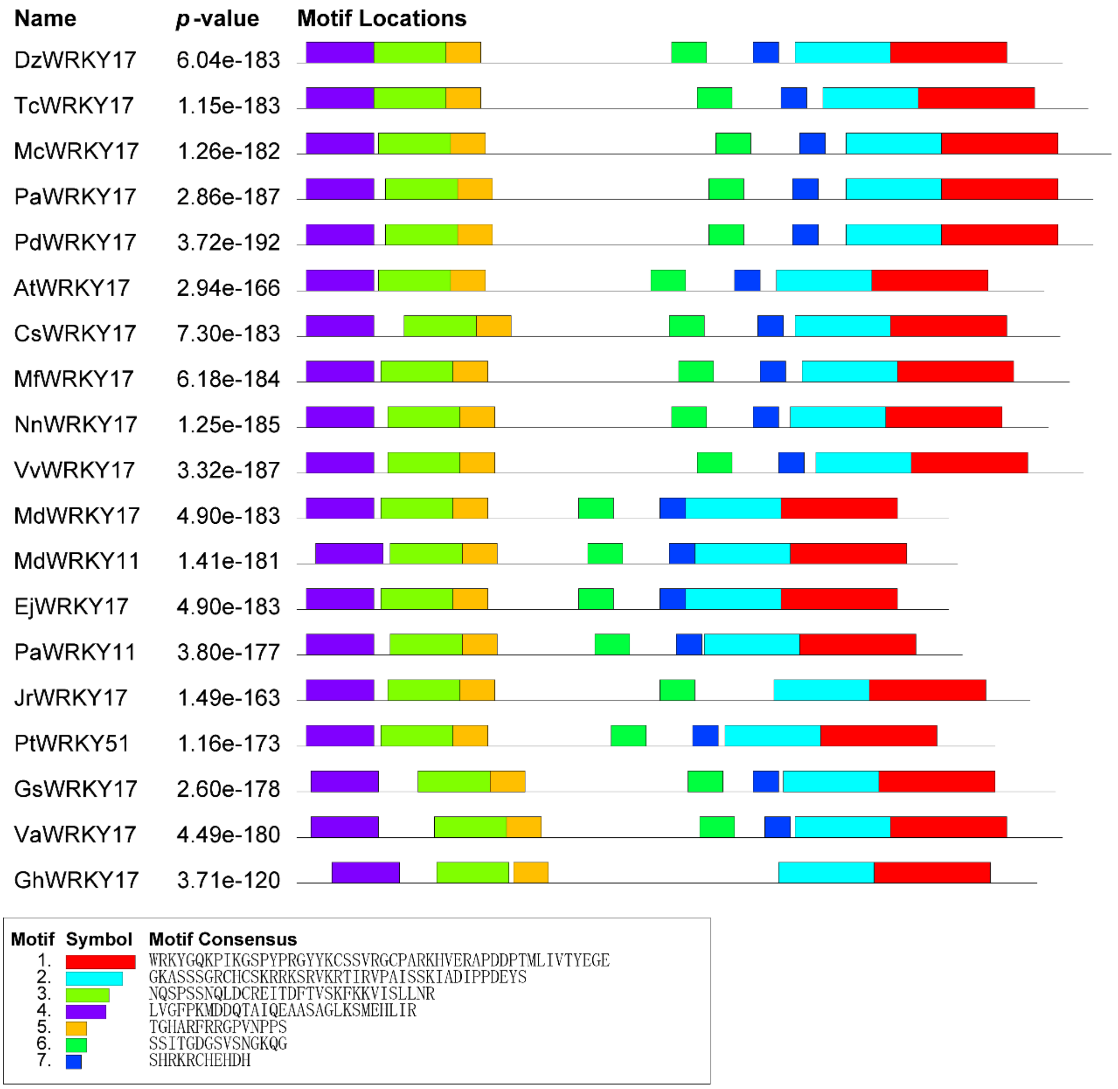

Figure S1 The putative conserved motifs in WRKY proteins

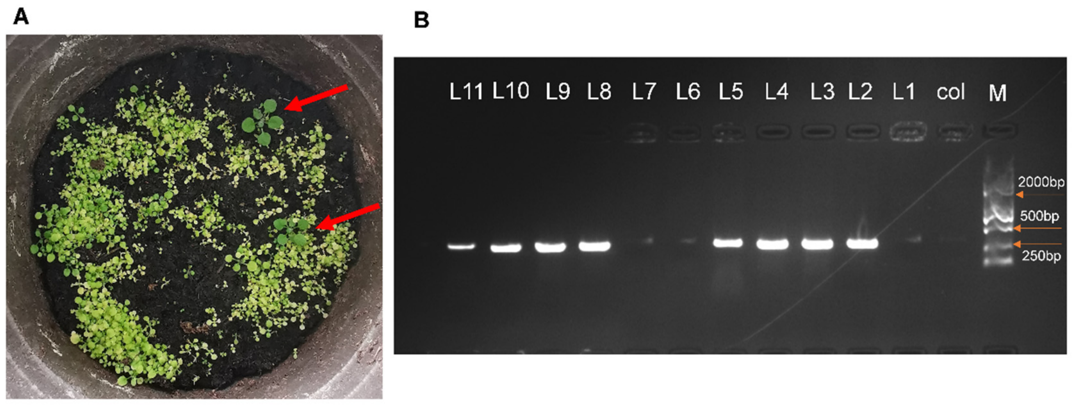

Figure S2 Selection of *A. thaliana* seeds in presence of glufosinate-ammonium (A) and PCR(B).
Note: Col: genome DNA of wild type; L1-L11: pFGC5941 plasmid DNA

Table S1 Putative cis-acting elements identified in the promoter region of *EjWRKY17* gene

Cis-acting elements	Number	Function
3-AF1 binding site	1	light responsive element
AAGAA-motif	2	MeJA-responsiveness
ABRE	1	cis-acting element involved in the abscisic acid (ABA) responsiveness
AE-box	1	part of a module for light response
ARE	1	cis-acting regulatory element essential for the anaerobic induction
AT~TATA-box	2	core promoter element around -30 of transcription start
AuxRR-core	1	cis-acting regulatory element involved in auxin responsiveness
Box 4	3	part of a conserved DNA module involved in light responsiveness
CAAT-box	53	common cis-acting element in promoter and enhancer regions
CCAAT-box	1	MYBHv1 binding site
CGTCA-motif	1	cis-acting regulatory element involved in the MeJA-responsiveness
DRE core	1	dehydration responsive element
ERE	2	ethylene-responsive element
G-Box	1	cis-acting regulatory element involved in light responsiveness
GA-motif	1	part of a light responsive element
GATA-motif	1	part of a light responsive element
GCN4_motif	1	cis-regulatory element involved in endosperm expression
LTR	1	cis-acting element involved in low-temperature responsiveness
MYB	3	Drought related
MYB recognition site	1	Anthocyanin transport regulation
MYB-like sequence	1	GA and Sugar regulation (α-amylase)
MYC	10	Associated with the dehydration response
MBS	1	MYB-binding site
O2-site	1	cis-acting regulatory element involved in zein metabolism regulation
P-box	1	gibberellin-responsive element
STRE	5	Oxidative stress responsive element
TATA-box	21	core promoter element around -30 of transcription start
TC-rich repeats	1	cis-acting element involved in defense and stress responsiveness
TCCC-motif	1	part of a light responsive element
TGACG-motif	1	cis-acting regulatory element involved in the MeJA-

		responsiveness
W box	1	elicitor-responsive element
WRE3	2	wound-responsive element
as-1	1	involved in shoot-specific expression and light responsiveness
circadian	1	cis-acting regulatory element involved in circadian control

Table S2 Prediction of WRKY binding cis-elements in the promoter of stress-responsive genes

Gene	WRKY protein binding sites	Number
ABF1	TTGACC	1
RD29A	TTGACC/TTGACT	3
RD29B	TTGACT	1
RD22	TTGACT	3
RAB18	TTTTCCAC	2
LEA14	TTGACC/TTGACT	5
LEA76	TTGACC	2
KIN1	TTGACT	4

Table S3 Primers used for gene cloning

Primer name	Primer sequences（5′-3′）	Purpose of primers
WRKY17-F	TGCAAGAAGCTGCCTCCGC	partial sequences forward primer
WRKY17-R	GCTCTCTCCACGTGTTTCCTTGC	partial sequences reverse primer
GSPWRKY17-F1	CCGGCTTTCTCCGCTGGAAAACC	3′RACE gene specific outer primer
GSPWRKY17-F2	GGTCAAAAGCCGATCAAGG	3′RACE gene specific inner primer
GSPWRKY17-R1	GCTTGAACTTGGTGACGGTGAA	5′RACE gene specific long primer
GSPWRKY17-R2	AGGGTGCGGATGAGGTGGTG	5′RACE gene specific short primer
EjWRKY17-F	ATGGCTGTAGATCTAGTTGGCTTCTC	full-length forward primer
EjWRKY17-R	TCATTCTTTAGAAGATTGGAAAACAAGGC	full-length reverse primer

Table S4 Names and accession numbers used for phylogenetic analysis

Name	Species	Accession number
DzWRKY17	*Durio zibethinus*	XP_022731890.1
TcWRKY17	*Theobroma cacao*	XP_007011614.2
McWRKY17	*Momordica charantia*	XP_022141783.1
PaWRKY17	*Prunus avium*	XP_021834784
PdWRKY17	*Prunus dulcis*	XP_034200082
AtWRKY17	*Arabidopsis thaliana*	AT2G24570.1
CsWRKY17	*Camellia sinensis*	XP_028113091.1
MfWRKY17	*Myrothamnus flabellifolia*	QNN83752.1
NnWRKY17	*Nelumbo nucifera*	XP_010265415.1
VvWRKY17	*Vitis vinifera*	XP_002262775.1
MdWRKY17	*Malus domestica*	ADL36859.1
MdWRKY11	*Malus domestica*	XP_028951936.1
PaWRKY11	*Prunus avium*	XP_021815841.1
JrWRKY17	*Juglans regia*	XP_018859253.1
PtWRKY51	*Populus trichocarpa*	XP_002325248.2
GsWRKY17	*Glycine soja*	XP_028197771.1
VaWRKY17	*Vigna angularis*	XP_017410351.1
GhWRKY17	*Gossypium hirsutum*	ADW82098.1

Table S5 Primers used for vector construction

Primer name	Primer sequences（5′-3′）	Primer sequences（5′-3′）
pFGC5941 vector	aggcgcgccATGGCTGTAGATCTAGTTGGCTTCTC(AscI)	gctctagaTCATTCTTTAGAAGATTGGAAAACAAGGC(XbaI)
pCAMBIA1300-GFP vector	cgcggatccATGGCTGTAGATCTAGTTGGCTTCTC (BamHI)	gctctagaTTCTTTAGAAGATTGGAAAACAAGGC (XbaI)
CaMV 35s	TGAGACTTTTCAACAAAGGATAATT	TGTCCTCTCCAAATGAAATGAAC

Table S6 Primers used for quantitative real-time PCR (qRT-PCR)

Primer name	Primer sequences（5′-3′）	Primer sequences（5′-3′）
qEjWRKY17	AAAAGCCGATCAAGGGCTCA	TGGAAAACAAGGCCCACCAC
qEjActin	AATGGAACTGGAATGGTCAAGGC	TGCCAGATCTTCTCCATGTCATCCCA
AtActin	TTACCCGATGGGCAAGTC	GCTCATACGGTCAGCGATAC
AtABF1	GAGACTAGCGCAGATGGTCC	CTAGCAGCGGATTCCCGATT
AtRD29a	AGGAGGAATGGTTGGGAGGA	TTCTGCACCGGAACAACAGT
AtRD29b	ACGTCGTTGCCTCAAAGCTA	TTGCGTCTCCTTCACTCCAC
AtRD22	GGTTCGGAAGAAGCGGAG	GAAACAGCCCTGACGTGATAT
AtCOR15A	GGCCACAAAGAAAGCTTCAG	CTTGTTTGCGGCTTCTTTTC
AtRAB18	TCGGTCGTTGTATTGTGCTTTTT	CCAGATGCTCATTACACACTCATG
AtLEA14	GTCATTCGATTCCGATCTGTGAGATC	GTCATTCGATTCCGATCTGTGAGATC
AtLEA76	GGTGAAGCACACTTTAGGGC	TTCCTCTGTGTCTCACGAGTAGT
AtKIN1	AACAAGAATGCCTTCCAAGC	CGCATCCGATACACTCTTTCC
